# Stability of Ensemble Models Predicts Productivity of Enzymatic Systems

**DOI:** 10.1371/journal.pcbi.1004800

**Published:** 2016-03-10

**Authors:** Matthew K. Theisen, Jimmy G. Lafontaine Rivera, James C. Liao

**Affiliations:** 1 Department of Bioengineering, University of California, Los Angeles, Los Angeles, California, United States of America; 2 Department of Chemical and Biomolecular Engineering, University of California, Los Angeles, Los Angeles, California, United States of America; 3 UCLA-DOE Institute, University of California, Los Angeles, Los Angeles, California, United States of America; Georgia Institute of Technology, UNITED STATES

## Abstract

Stability in a metabolic system may not be obtained if incorrect amounts of enzymes are used. Without stability, some metabolites may accumulate or deplete leading to the irreversible loss of the desired operating point. Even if initial enzyme amounts achieve a stable steady state, changes in enzyme amount due to stochastic variations or environmental changes may move the system to the unstable region and lose the steady-state or quasi-steady-state flux. This situation is distinct from the phenomenon characterized by typical sensitivity analysis, which focuses on the smooth change before loss of stability. Here we show that metabolic networks differ significantly in their intrinsic ability to attain stability due to the network structure and kinetic forms, and that after achieving stability, some enzymes are prone to cause instability upon changes in enzyme amounts. We use Ensemble Modelling for Robustness Analysis (EMRA) to analyze stability in four cell-free enzymatic systems when enzyme amounts are changed. Loss of stability in continuous systems can lead to lower production even when the system is tested experimentally in batch experiments. The predictions of instability by EMRA are supported by the lower productivity in batch experimental tests. The EMRA method incorporates properties of network structure, including stoichiometry and kinetic form, but does not require specific parameter values of the enzymes.

## Author Summary

A method of metabolic simulation called ensemble modelling for robustness analysis is used to predict the behavior intrinsic to the network structure (stoichiometry and kinetic form) of four enzymatic systems. Some network structures are shown to be prone to instability. Starting from a stable system, instability is also predicted to be caused by increasing amounts of certain enzymes. EMRA is a valuable tool for pathway design, particularly synthetic pathways which are uncontrolled and not stabilized through evolution.

## Introduction

**Fig 1:**
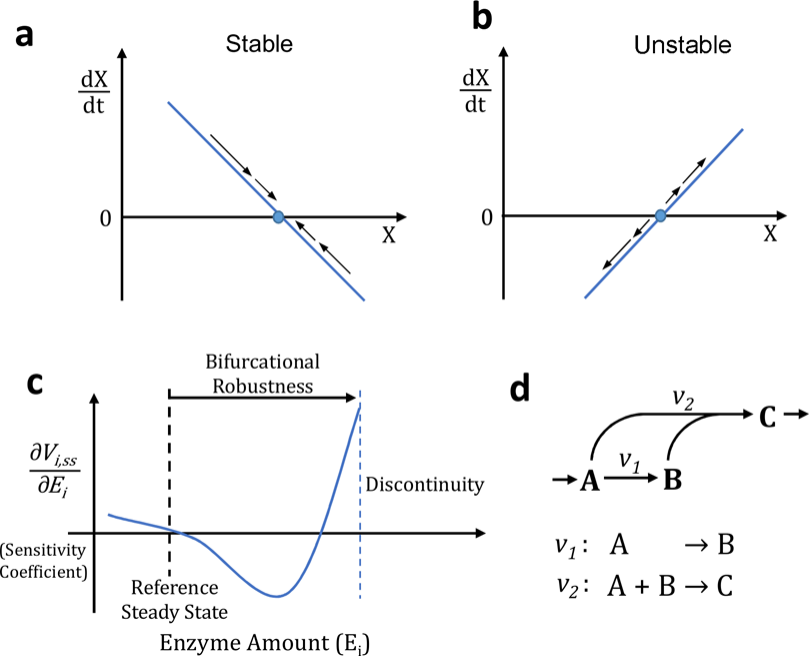
Schematic figure showing how instability can occur and how it can cause lower production in batch experiments. **a-b)** In a one-dimensional dynamical system, the sign of d**ẋ**/d**x** determines stability of a fixed point (**ẋ** = 0). If the sign of d**ẋ**/d**x** is negative **(a)**, the system is stable to stochastic perturbations from the fixed point. In contrast, if d**ẋ**/d**x** positive **(b)**, the fixed point is unstable. In a multivariate system, the analogous value is the maximum of the real parts of the eigenvalues of the Jacobian matrix. (i.e. if max(Re(Eig(Jac)))) is greater than 0 (the jacobian is singular), the fixed point will be unstable, if it is less than zero the fixed point will be stable unstable). **c)** Traditional global sensitivity analysis calculates the sensitivity coefficient which represents the derivative of steady state production with respect to enzyme amount. However, sensitivity analysis doesn’t investigate the likelihood of instability. Bifurcational robustness measures the distance between the reference steady state and the bifurcation point. **d)** A kinetic trap in which multiple reactions (v_1_ & v_2_) are competing for the same substrate (A). If the enzyme catalyzing v_1_ increases greatly, it may cause instability by decreasing [A] so much that v_2_ cannot continue.

Metabolic systems typically operate either under a stable steady state or an oscillatory mode. A non-oscillatory unstable system may result in multiple problems, including depletion of metabolites essential for growth, accumulation of toxic intermediates, or depletion of cofactors in the pathway—all ultimately leading to loss of production or cell death. While systems with stable steady states or sustained oscillation have been studied extensively [1–6], to our knowledge metabolic systems prone to instability have not been investigated as much. Both stable (Fig 1A) or unstable (Fig 1B) system have a mathematical steady state (or fixed point), but the unstable steady state is not realizable in the physical world because any deviations from the steady state are amplified. Therefore, through evolution the unstable systems are selected against or stabilized by various levels of controls. However, the issue of stability is particularly important when engineering a novel pathway or altering an existing one.

Furthermore, even starting from a stable steady state system, increasing an enzyme activity beyond a specific level may result in system failure (see Fig 1C) because the system enters an unstable region, resulting in loss of a productive steady state. The likelihood of losing stability is characterized by bifurcational robustness using Ensemble Modeling for Robustness analysis (EMRA) [7]. Instability caused by enzyme perturbation has been predicted in proposed synthetic pathways and natural pathways in previous analyses[7,8]. One means of stability loss, among other possibilities, is a kinetic trap (Fig 1D), resulting from a metabolic branch point within a cyclic pathway. Upon perturbation, a kinetic trap may cause a sudden, unexpected, and qualitative change in dynamic behavior (Fig 1C). Since cyclic pathways are common in metabolism, particularly when cofactor recycling are involved, such examples are copious. The bifurcational robustness is a measure of how far an enzyme amount must be perturbed before bifurcation occurs (Fig 1C). Sudden system failure due to entering an unstable regime differs from the gradual deterioration of performance characterized by local sensitivity analysis. Sensitivity analysis, Biochemical Systems Theory [9–13], or metabolic control analysis (MCA) [14] is concerned with identifying the sensitivity coefficient (Fig 1C), which is the derivative of steady state production flux with respect to enzyme amount. In this work, we further examine the tendency for a metabolic system to be unstable based on their intrinsic network structure, which is determined by the network stoichiometry and kinetic rate laws. One way that this work builds on global sensitivity analysis is in that it focuses heavily on what we term the bifurcational robustness (Fig 1C), rather than the value of the sensitivity coefficient.

In previous uses of EMRA, unstable parameter sets found while constructing ensembles were discarded [7,8]. Here, we examine the intrinsic probability for a system to be unstable. This is fundamentally distinct from the tendency to bifurcate upon change from a stable steady state. In addition, previous EMRA simulations were applied to continuous processes. However, production experiments using enzymatic systems—whether *in vitro* or *in vivo*—are often carried out as a batch system due to practical considerations. Thus, it is unclear how simulations from a continuous mode can inform experimental strategies for new metabolic pathways which are investigated in batch or cell free experiments. Using four metabolic systems, we showed that the instability problem discussed above is indeed an issue, even with batch experiments. Interestingly, this type of abrupt change is observed in common biological systems, including glycolysis. The results suggest that the stability issues may be more prevalent than previously appreciated.

### Systems description

We use the following enzymatic systems as examples for our investigation. Three of these systems have been described previously and some experimental data are available to validate our predictions. The other system (glucose to isoprene pathway) has not been experimentally investigated.

### Methanol Condensation Cycle (MCC)

**Fig 2:**
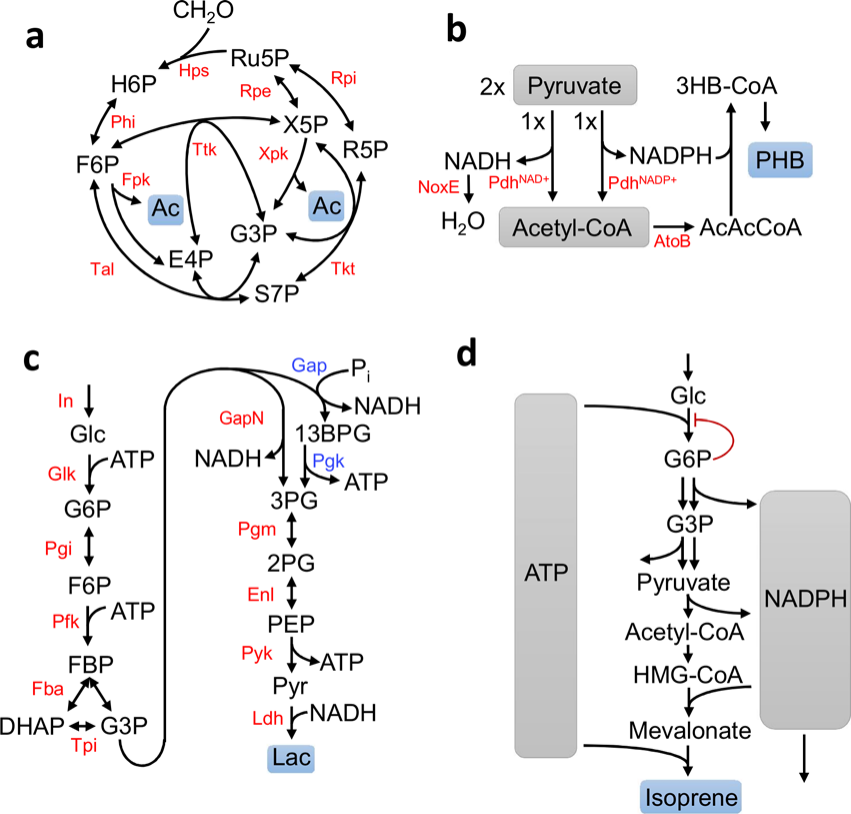
Schematics showing four enzymatic systems which can be investigated by EMRA. **a)** A methanol condensation cycle (MCC) which converts formaldehyde to acetyl-phosphate with 100% carbon efficiency. Acetate can be generated enzymatically. **b)** A molecular purge valve which dissipates reducing power in order to convert pyruvate to polyhydroxybutyrate (PHB) in a redox balanced way. **c)** A chimeric glycolysis system which converts glucose to lactate in a redox- and ATP-balanced route. It uses a non-phosphorylating GapN to maintain ATP balance. The corresponding route through standard Embden-Meyerhof-Parnas (EMP) glycolysis is shown with blue enzyme labels. **d)** Glucose to isoprene pathway which uses NADPH-dependent glyceraldehyde-3-phosphate dehydrogenase (GAPDH) and pyruvate dehydrogenase (PDH). An NADPH drain is required to maintain redox balance. This pathway is also ATP-balanced. G6P inhibition is also considered in this system.

Methanol condensation cycle (MCC) (Fig 2A) is a metabolic pathway to convert methanol to higher alcohols with 100% theoretical carbon yield, in contrast to natural pathways like ribulose monophosphate (RuMP) which have a maximum of 67% theoretical carbon yield due to the decarboxylation of pyruvate [15]. The core of the pathway creates a C-C bond between two formaldehyde molecules derived from methanol for the generation of acetyl-phosphate, which can be enzymatically converted to acetate or ethanol.

In this cycle, formaldehyde is incorporated into ribulose-5-phosphate (Ru5P) (Fig 2A) to generate hexulose-6-phosphate (H6P) by hexulose phosphate synthase (Hps). H6P is then isomerized to fructose-6-phosphate (F6P) which can be cleaved by phosphoketolase. Erythrulose-4-phosphate (E4P) and F6P can then recombine via transaldolase, transketolase and isomerases (Tal, Tkt, Rpe, Rpi) to regenerate Ru5P. Alternately, xylulose-5-phosphate (X5P) can be cleaved by phosphoketolase, yielding G3P and acetyl-phosphate. G3P is then shuffled with F6P by transketolase to generate E4P and X5P, which can proceed to regenerate Ru5P via Tal, Tkt, Rpe, and Rpi. The X5P- and F6P-cleaving activities of phosphoketolase are referred to as Xpk and Fpk, respectively, and the pathway is investigated with different combinations of these activities.

### Pyruvate to poly-hydroxybutyrate

A molecular purge valve for the production of polyhydroxybutyrate from pyruvate *in vitro* was demonstrated by Opgenorth *et al* (Fig 2B) [16]. This system needs special attention to achieve redox balance, since pyruvate has a more reduced oxidation state than the product. To alleviate this cofactor imbalance, a method for dissipating excess reducing equivalents, termed a molecular purge valve, was designed for the conversion of pyruvate to downstream products like isoprene and poly(hydroxybutyrate) (PHB). Two different pyruvate dehydrogenases (PDH) were used in the system—one with cofactor specificity for NADPH and one with specificity for NADH. The downstream pathway enzymes use NADPH to reduce metabolites and an NADH oxidase (NoxE) to dissipate the generated NADH. From two acetyl-CoA molecules, two enzymes are required to generate the final product PHB.

### A chimeric ATP-balanced glycolysis system

A chimeric glycolysis system was demonstrated by Ye *et al* [17] (Fig 2C). Canonical Embden-Meyerhof-Parnas (EMP) glycolysis generates a net of two ATP per glucose. In the chimeric system, a non-phosphorylating glyceraldehyde-3-phosphate dehydrogenase (GAPN) was used. This results in a system which is ATP balanced, making it more convenient for *in vitro* assays. Additionally, the system is NADH balanced since the final product was lactate, which has the same redox state as glucose.

### Glucose to isoprene system

A system is considered for the conversion of glucose to isoprene (Fig 2D). NADPH-dependent glyceraldehyde-3-phosphate dehydrogenase (GAPDH) and NADPH-dependent pyruvate dehydrogenase are used in the pathway. NADPH is used since the downstream reactions in isoprene synthesis use NADPH. The pathway converts three glucose to two isoprene molecules. Interestingly, this pathway is also ATP-balanced, with the ATP generated by the glycolytic pathway being used stoichiometrically downstream in the isoprene pathway reactions. However, to maintain redox balance, NADPH must be drained from the system, potentially via an oxidase or similar enzyme. This system is investigated both with and without a substrate-level regulation of glucokinase (GK) by G6P, implemented using an irreversible version of modular rate laws [18] proposed by Liebermeister. The kinetic form used is known as competitive inhibition, though many other kinetic forms are plausible. Inhibition of this step by G6P is well-known. For example, a human enzyme catalyzing this reaction is G6P-inhibited [19]. These equations show the effective kinetic forms of glucokinase used without and with regulation:

$$No\;Regulation:\;V_{GK}=\frac{V_{max}}{\frac{K_{m,Glc}}{\left[Glc\right]}+\frac{K_{m,ATP}}{\left[ATP\right]}+\frac{K_{m,ATP}K_{m,Glc}}{\left[ATP\right]\left[Glc\right]}+1}$$

$$With\;G6P\;Inhibition\;V_{GK}=\frac{V_{\text{max}}}{\frac{K_{m,Glc}}{\left[Glc\right]}+\frac{K_{m,ATP}}{\left[ATP\right]}+\frac{K_{m,ATP}K_{m,Glc}}{\left[ATP\right]\left[Glc\right]}\left(1+\frac{\left[G6P\right]}{K_{i,eff,ATP}}\right)+1}$$

## Results

### Network intrinsic stability differs among systems and is different from bifurcational robustness upon perturbation

We used the four systems described in Fig 2 to examine the stability problem. In particular, we investigated how network structure affects the intrinsic possibility of reaching stability. Previous EMRA work starts from an ensemble of parameter sets that give the same reference steady state, and discards the parameter sets that generate a Jacobian matrix with a real part of an Eigenvalue greater than zero, which indicates instability.

**Fig 3:**
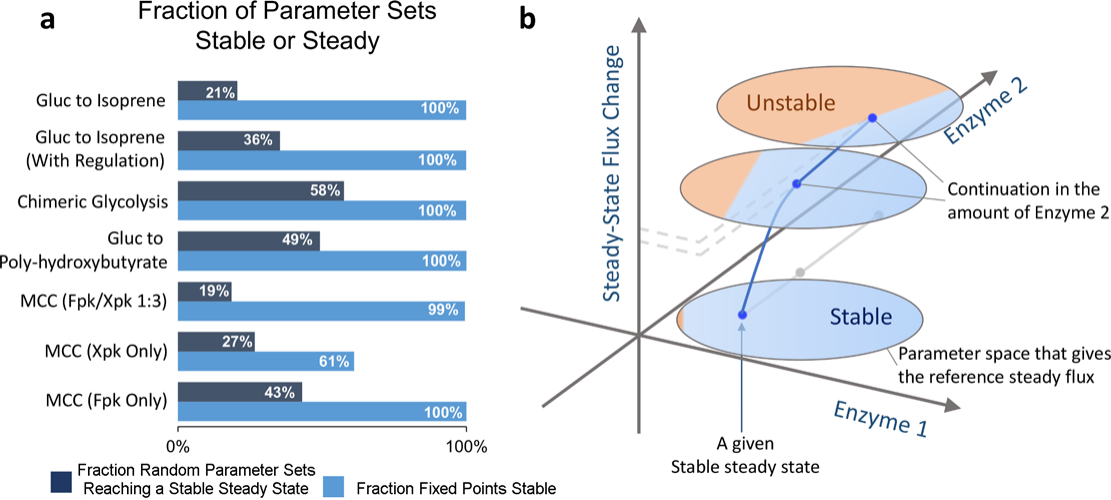
Characterizing intrinsic stability of different pathway systems. **a)** For different pathways, two measures of intrinsic stability are presented. First, in dark blue, is the fraction of unconstrained, random parameter sets which reach a productive steady state. Second, in light blue, is the fraction of EMRA-determined parameter sets constrained to a steady state which are also stable. The intrinsic stability of pathways differs greatly between pathways, and also depending on which measure is used. Thus, a rational method of pathway balancing would be useful. (SD < 2% for all systems, n = 3 x 1000 parameter sets). Since phoshoketolase has two activities, cleaving either F6P (called Fpk) or X5P (called Xpk), we investigated used a ratio of Fpk/Xpk activites, 1:3. **b)** A representation of how steady state is not always stable. After perturbation from a constrained steady state, the fraction of parameter sets which retain stability tends to decrease, and steady state flux may change. Eventually, a parameter set may become unstable after perturbation.

However, experimental systems are not guaranteed to be stable or reach a steady state. To place stability and steady state in a context which is more meaningful to experimental efforts, enzyme parameters were chosen completely at random, and the systems were then integrated in time domain to determine if a productive steady state was reached (Fig 3A, dark blue bars). This method is more representative of experimental efforts which often have either little or indirect control over enzyme amount or activity (*in vivo*), or don’t have rational methods for pathway balancing (both *in vivo* and *in vitro*). Interestingly, the results show that pathways have very different likelihoods of resulting in a steady state (Fig 3A, dark blue). The glucose to isoprene system had only 21% of randomly generated parameter sets reaching a non-trivial (non-zero) steady state. This could be because it is a relatively large system in terms of enzyme number and uses two different cofactors (NADPH and ATP). A large system may be less likely to reach a steady state. If each enzyme has an acceptable range of values, then in a large system it is more likely that at least one of these values would be outside the range, resulting in system instability. However, when regulation of glucokinase was introduced via activation by ATP and inhibition by ADP, the likelihood of productivity jumped to 36% (Fig 3A). Overall, these results show that intrinsic pathway structure and kinetic forms (including regulation) have a strong influence on possibility of reaching a productive steady state. The result is a varying, and sometimes low, likelihood of achieving stability and productivity. Thus, finding rational ways to balance pathways is an important goal which can improve and accelerate the pathway development process.

If the enzyme parameters were first constrained to a fixed point by solving for parameter values which give reaction rates equal to the reference flux, then the probability of attaining stability is greatly increased. While this is not practical in experiments, the method proves useful in model construction. We found that (Fig 3A, light blue bars) the fraction of fixed points which were stable varied depending on the network structure. While most systems showed at least 99% of the parameter sets sampled to be at a stable steady state, the MCC (Xpk-only) system showed only 61% of parameter sets to be stable. Although for some systems the fraction of stable steady states is similar—5 systems which all show at least 99% stability by this measure—they have varying tendency to lose stability upon perturbation (Fig 3B). Starting from the reference state, where parameter sets are chosen under the fixed point constraint, the region of instability could grow when enzyme parameter changes (Fig 3B). Depending on the structure of the system, the instability region might grow in a different fashion upon perturbation, and eventually some might lose stability. This shows that stability of fixed points in metabolic systems is not guaranteed and that stability could be a critical factor in metabolic systems.

### Stability of continuous systems upon perturbation can inform results of batch systems

EMRA uses continuous production models to simulate enzymatic systems. However, many experiments, including *in vivo* and *in vitro*, are conducted as batch processes. Thus, it’s not clear how a perturbation which causes bifurcation in a continuous system will inform the batch experiment. Where a pseudo-steady state may exist, the pseudo-steady state behavior can be predicted by the continuous model. In these systems, if a parameter set resides in a domain where no stable steady state exists in the continuous mode, then no stable pseudo-steady state exists in the batch mode. This can be justified by locally linearizing the input function to convert a pseudo-steady batch system to a continuous system. However, an experimentalist measuring only the product output at the end point would not detect the lack of stability. In this case, the product yield will gradually decrease even when the system has entered an instability region.

**Fig 4:**
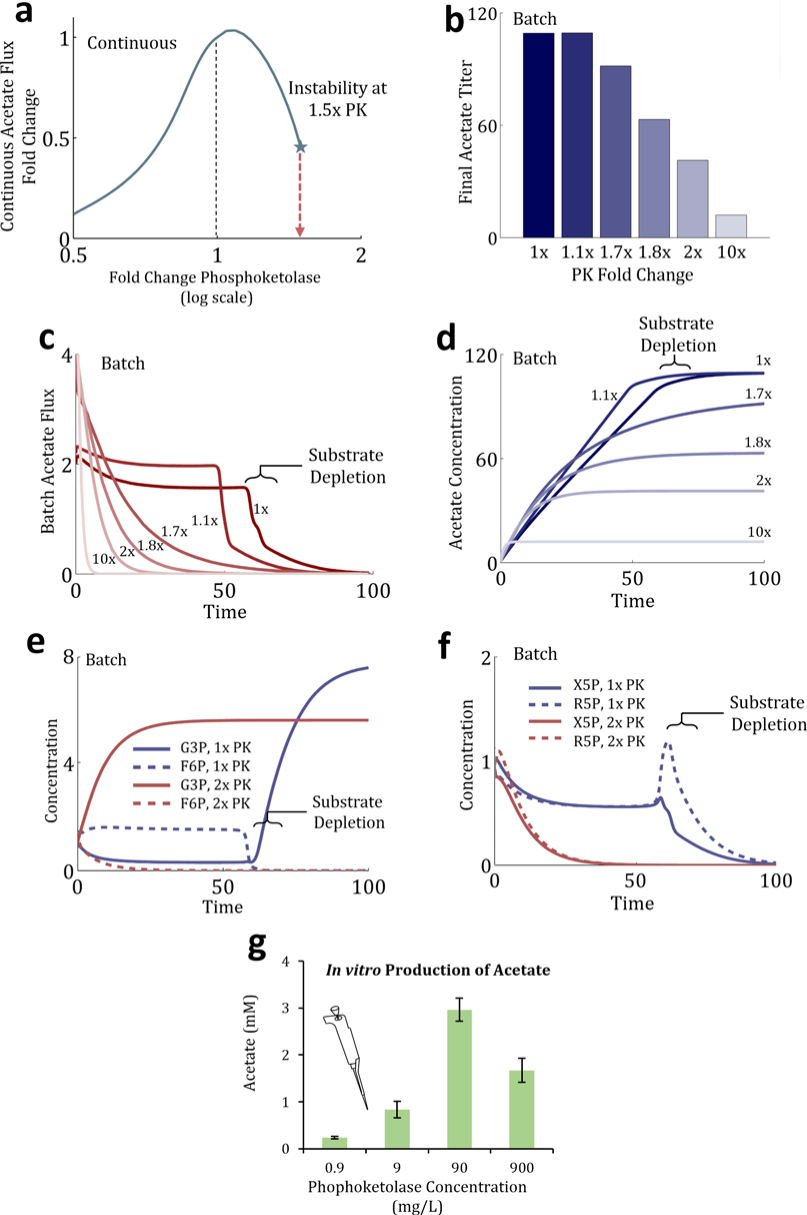
Investigating the instability in the MCC pathway using Fpk/Xpk ratio as 1:3. **a)** In a continuous system, an arbitrary parameter set determined by EMRA is perturbed up and down with respect to phosphoketolase, on the X-axis. On the Y-axis, the continuous, steady state, acetate flux is plotted. As phosphoketolase increases, the system bifurcates at ~1.5x increase. **b)** Time domain simulation is performed, at different amounts phosphoketolase (PK). The final titer for each condition is plotted. The production gradient appears gradual, but is the result of a sudden instability. **c)** The production rate for acetate is shown for each phosphoketolase amount over time. In the stable conditions (1x & 1.1x), production rate reaches a constant, implying the system enters a “pseudo-steady state”, until substrate depletes. In the other conditions, production rate is never steady, but decreases over time. **d)** The amount of acetate is plotted over time. It is observed that as the amount of phosphoketolase increases beyond bifurcation, the production decreases. **e)** At the 1x and 2x conditions, the concentrations of G3P and F6P are plotted. In the 1x condition, F6P is maintained at a nonzero-level throughout production, while in the 2x condition, it is quickly depleted and G3P accumulates. **f)** The R5P & X5P levels are plotted with time in the 1x and 2x conditions. **g)** Data from Bogorad *et al* [15] shows that as phosphoketolase level increased, the amount of acetate produced by the cycle decreased, supporting a link between instability in a continuous system and production in an analogous batch system. An icon shows this data is experimental.

To show how the existence of a continuous bifurcation could manifest itself in a batch system, we simulated a batch system in time domain. First, stable parameter sets were generated via EMRA in a continuous MCC system using Fpk/Xpk ratio as 1:3. Then, the parameter sets were integrated using the continuation method to increase the phosphoketolase level until instability occurs, increasing V_max_ for Fpk & Xpk at the same ratio. A representative parameter set is plotted in Fig 4A to show the effect of increased phosphoketolase on continuous steady state acetate flux up to the point of instability. As phosphoketolase increases, the flux towards product increases slightly before decreasing and finally becoming unstable.

This parameter set was found to become unstable at a ~1.5-fold increase of phosphoketolase. Different amounts of phosphoketolase perturbation (1x, 1.1x, 1.7x, 1.8x, 2x, 10x –multiplier applied to both Fpk & Xpk V_max_ values) were chosen to show the dynamic response of the system in a batch simulation. All rate equations, parameter values and initial conditions were kept the same as in the continuous model (i.e. all starting metabolite concentrations were normalized to unity), except that starting formaldehyde concentration was multiplied by 200 and the “in” and “out” reactions used in the continuous mode were eliminated to observe product accumulation in time domain simulation of the batch system. 200-fold increase in initial formaldehyde concentration was chosen arbitrarily to signify a batch reaction, in which the starting substrate was included as a single charge instead of being fed over time. It was found to adequately demonstrate the phenomena we were interested in, though other values could have worked as well. See Tables 1 and 2.

**Table 1:** Summary of difference between flux equation parameter values in continuous and batch simulations of the MCC system (Fig 4).

	Formaldehyde Input Exchange Rxn, V_max_	Acetate Output Exchange Rxn, V_max_	All Other V_max_	$\bar{\lambda }$ (K_m,_ K_eq_, etc.)
Continuous	Constrained to Reference Input Flux	Constrained to Reference Output Flux	Constrained to Reference Flux	Selected from random distribution
Batch	0	0	Same values as above	Same values as above

**Table 2:** Comparison of normalized metabolite concentration values in continuous and batch simulations performed on the MCC system (Fig 4).

	CH_2_O	Ru5P	H6P	F6P	E4P	S7P	G3P	X5P	R5P	AcP
Normalized *Continuous Steady State* Metabolite Concentration, ${\bar{\text{X}}}_{\text{i}}$	1	1	1	1	1	1	1	1	1	1
Normalized *Batch Initial* Metabolite Concentration, ${\bar{\text{X}}}_{\text{i}}$	200	1	1	1	1	1	1	1	1	1

Interestingly, the final batch production observed for this system decreases gradually as phosphoketolase (PK) amount (Fig 4B) increases. In the continuous system, the underlying phenomenon is instability, a step change in the nature of the steady state. In the corresponding batch system, pseudo-steady state disappears because of instability. However, the product formation does not stop until key intermediates are depleted. Batch acetate production rate over time is plotted in Fig 4C. For the stable 1x and 1.1x conditions, a pseudo-steady state was achieved in which acetate production rate reached a constant level, only decreasing when the formaldehyde had been consumed. However, for the conditions which are past the instability point (1.7x – 10x), a steady rate of acetate production is never achieved. Instead, the rate decreases monotonically until it reaches zero. The productivity of the 10x condition falls the fastest, eventually resulting in the lowest production. This shows that a decrease in production, even gradually, in a batch system could be associated with an instability issue in an analogous continuous system. In Fig 4D, the acetate concentration over time is plotted to show how the system evolves over time.

For systems that are stable, because the initial concentration of the starting substrate is much higher than the K_m_ value of the uptake system, the rate of input holds largely constant until the substrate concentration approaches the Km value. During this time, the system is operating under a pseudo-steady state similar to a stable continuous system. This is seen in Fig 4 for 1x and 1.1x phosphoketolase concentrations. Thus, the property of continuous system simulation carries over, until substrate concentration approaches K_m_. Thus, the system is run almost the same as in a continuous system in the first 50 min time units or so (Fig 4C), when most of the acetate is produced (Fig 4D)

For systems that are unstable (Fig 4, 1.7x, 1.8x, 2x, 10x phosphoketolase concentrations), the output flux was not able to reach a steady-state (Fig 4C), and it decreases rapidly from the start and approaches zero despite the presence of the initial substrate. The cumulative product formed (acetate) is the integral of flux over time (Fig 4D), which decreases as the system moves further away from the bifurcation point.

Additionally, we investigated the mechanism by which the bifurcation causes decreased production. In the 1x condition, F6P is maintained at a nonzero-level throughout production, while in the 2x condition, it is quickly depleted (Fig 4E). R5P and X5P are also shown to deplete quickly in the 2x condition (Fig 4F). Thus, it is the depletion of these cycle intermediates which causes cycle failure. A previous experimental effort ([20], data reproduced in Fig 4G) showed that in *in vitro* enzymatic experiments, the batch production of acetate with from formaldehyde reached a local maximum with respect to phosphoketolase amount, supporting the EMRA analysis. In sum, EMRA could potentially have useful insights into experimental systems, by identifying enzymes which may be most sensitive to bifurcation, and how they affect the system in question.

### EMRA predicts behavior of a molecular purge valve

**Fig 5:**
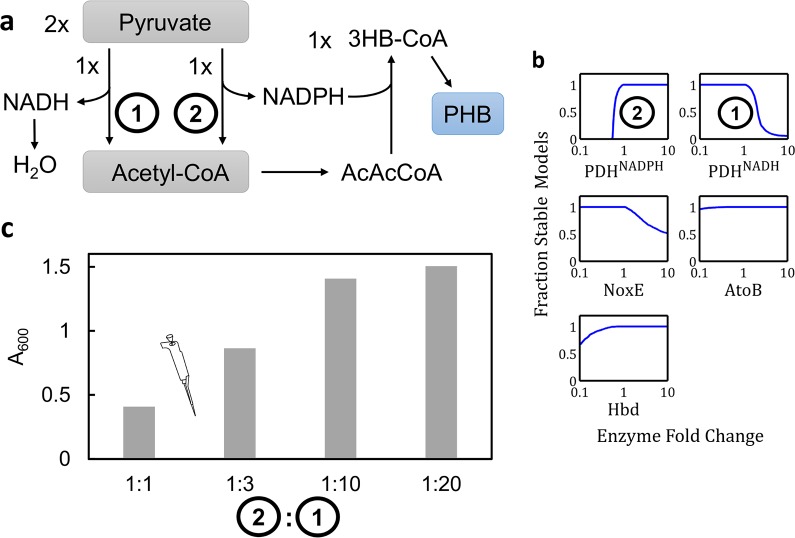
Stability of a biosynthetic purge valve for production of isoprenoids by dissipation of reducing equivalents. **a)** Pathway schematic showing cofactor requirements. **b)** Stability profiles predicted by EMRA (n = 1000) as enzyme amounts vary. It is shown that to maintain stability, high levels of PDH^NADP^ and low levels of PDH^NAD^ are required. **c)** Data from ([17], Fig 4) which shows that a high ratio of PDH^NADP^: PDH^NAD^ is required for optimal performance of the pathway. Image analysis of line graph figure from reference yielded numerical data to generate the bar graph shown. (1) indicates NADH-dependent PDH and (2) indicates NADPH-dependent PDH. An icon shows this data is experimental.

A purge valve system converting pyruvate to PHB was analyzed. Each enzyme is represented by a canonical Michaelis-Menten kinetic rate law, and the reference flux is fixed since there are no degrees of freedom. EMRA methodology was implemented in this system to show the effects of perturbation of each enzyme. High NADPH-dependent PDH (PDH^NADPH^) (Fig 5B) and low NADH-dependent PDH (PDH^NADH^) resulted in the most stability for the pathway. PDH^NADH^ must be low to prevent too much pyruvate from taking this route which generates unusable NADH reducing power, while PDH^NADPH^ must be high to ensure that enough NADPH is generated to allow for 100% yield from acetyl-CoA to PHB. The imbalance of these activities may cause system instability, according to EMRA. Indeed, the PHB pathway was experimentally demonstrated to have reduced production with a lower ratio of PDH^NADPH^:PDH^NADH^ [16] (Fig 5C), matching the results of EMRA.

### EMRA predicts chimeric glycolysis system’s sensitivity to glucose feed rate

**Fig 6:**
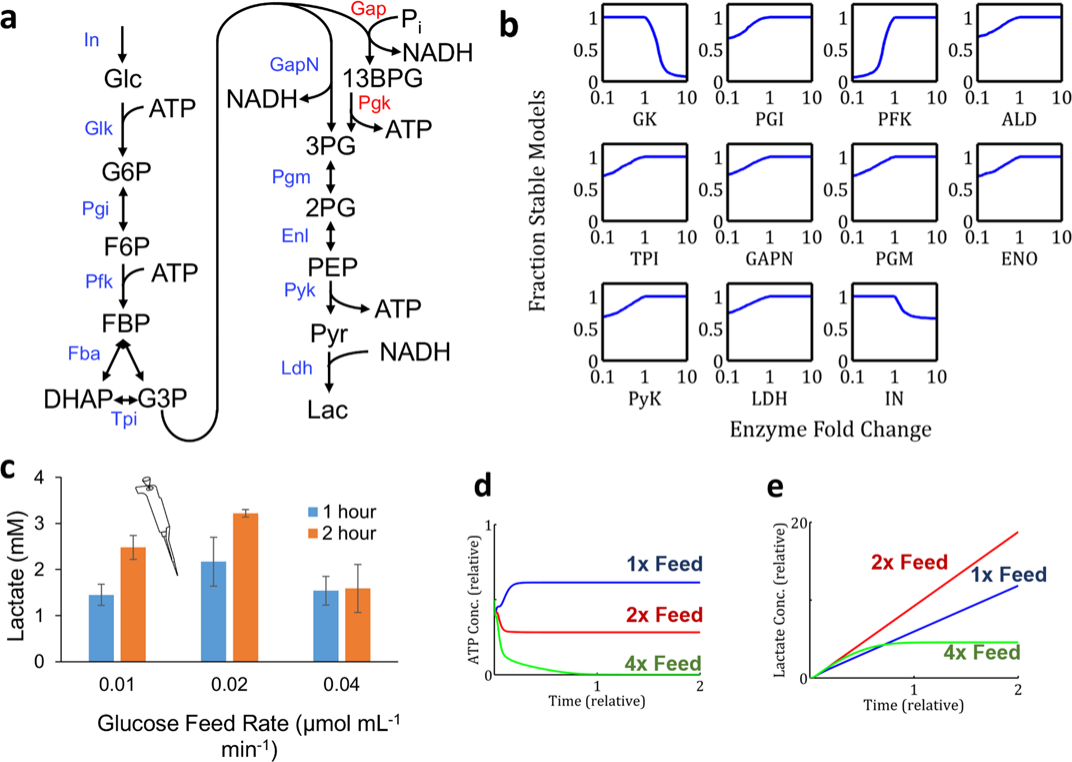
The ATP-balanced synthetic chimeric glycolysis pathway from glucose to lactate [17]. a) Pathway schematic contrasting cofactor production between standard Canonical Embden-Meyerhof-Parnas (EMP) glycolysis (Gap & Pgk, red lettering) with the chimeric non-phosphorylating GapN system. b) EMRA stability profiles (n = 1000) as enzyme amounts and glucose feed rate (IN) vary. Glucose feed rate is shown to produce moderate instability at higher levels. c) Data from (Fig 6A, [17]) which shows that increased glucose feed rate can cause lower production. An icon shows this data is experimental. d-e) Simulation of fed-batch production of a sample parameter set for the chimeric glycolysis system. Numerical integration of time domain behavior shows instability at higher feed rates caused by ATP depletion and resulting in lower overall lactate production. Priming intermediates are fed in the same proportion as the experimental condition, and feed rates are also demonstrated in the same proportion (1, 2, 4). d) ATP concentration over time at the three different glucose feed rates. e) Lactate production over time at three different glucose feed rates.

Another example of EMRA application is in a thermotolerant, cell-free glycolysis system which was demonstrated for the production of lactate from glucose by Ye *et al* (Fig 6A) [17]. Canonical Embden-Meyerhof-Parnas (EMP) glycolysis generates two net ATP per glucose, (Fig 6A, Gap & Pgk enzymes). However, in the chimeric system, to prevent cofactor imbalance, a non-phosphorylating glyceraldehade-3-phosphate dehydrogenase (GapN)[21] was used—resulting in a net balance of ATP and NADH from glucose to lactate. Again, the system was modeled using EMRA methodology. The results show that increased glucokinase (GK) amount and glucose feed rate (Fig 6B, GK, IN) may cause instability.

This system was experimentally tested for lactate production at different glucose feed rates (Fig 6C, data from Ye *et al* [17]). It was found that beyond a certain point, increasing glucose feed rate reduced lactate production, even if the same total amount of glucose had been fed, matching the instability to feed rate (IN) predicted by EMRA. The instability apparently occurs by the depletion of ATP by glucokinase. ATP is required for both glucokinase and phosphofructokinase (PFK). However, if glucose is fed too quickly, ATP may become depleted by glucokinase before it can be regenerated in lower glycolysis. A time domain simulation of this system was carried out using initial conditions similar to the experimental conditions reported [17] (Fig 6D and 6E) and a parameter set from EMRA which became unstable after increase of feed rate. The time domain simulation showed that at reference feed rate (1x), ATP level is maintained and lactate production continues. However, at 4x feed rate, the ATP is depleted and the lactate production stops.

EMRA was also carried out on canonical EMP glycolysis converting glucose to lactate and similar instabilities were found (S1 Fig). In both systems, reduction in PFK activity was shown to strongly increase chance of instability. This is because once a metabolite is past PFK, it may be used to regenerate ATP, so it’s important to ensure that the flux past PFK is sufficient to supply ATP for all of upper glycolysis. However, it’s more paradoxical that an increase in an enzyme would cause productivity and instability issues, particularly glucokinase, or even feed rate.

### EMRA predicts unstable enzymes in uncharacterized system from glucose to isoprene

**Fig 7:**
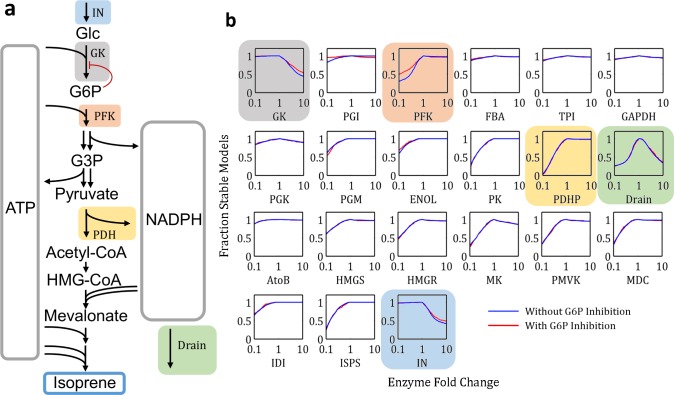
An NADPH-dependent pathway from glucose to isoprene. **a)** A pathway schematic showing an outline of the enzymatic reactions, cofactor flow, and regulation added (glucokinase). **b)** EMRA profiles for all (n = 1000) enzymes in the unregulated isoprene pathway in blue. EMRA profiles for all (n = 1000) enzymes in the GK-regulated isoprene pathway in red. Select enzymes are highlighted to show their position in the pathway. The enzymes dealing with ATP cycling are most changed by the presence of regulation.

To demonstrate the utility of EMRA in identifying potential points of instability, a not yet characterized pathway producing isoprene from glucose was investigated with EMRA (full pathway stoichiometry in S7 Table). The pathway is ATP-balanced and maintains redox balance using an NADPH drain. EMRA identifies that the NADPH drain must be balanced, not too low or too high. GK & IN must not be too high, while all other enzymes must only not be too low (Fig 7B). By introducing regulation of GK using modular rate laws [18], the fraction of productive steady states increased (Fig 3A). The stability to perturbation is also slightly improved for feed rate (IN), GK and PFK (Fig 7B).

Interestingly, the NADPH drain is unstable to both decrease and increase. This could be because if the rate is too low, then NADP^+^ is not sufficiently available for GAPDH, and lower glycolysis is unable to regenerate ATP needed for earlier in glycolysis and later in the pathway–while if the rate is too high, NADPH will not be available for the biosynthetic steps of the isoprene pathway. This analysis shows that a longer pathway has many complex, interacting factors that can cause instability and that EMRA is able to identify some of these potential issues. Similar to the previously simulated glycolysis system, glucokinase, (GK) and phosphofructokinase (PFK) and feed rate (IN) showed some instability. Other enzymes show less sensitivity upon increase (Fig 7B). Instability caused by decreasing enzyme concentration is common and seen in most if not all pathways.

A rational experimental plan for this pathway would thus focus on having sufficiently high levels of most enzymes (all except feed, glucokinase and drain), for example, by ensuring the total activity of each enzyme is significantly higher than the feed rate. Ensuring these enzymes are at a high level would ensure both stability, according to EMRA results, and the possibility of maximum productivity. For enzymes which become unstable at higher levels, more optimization is required. Levels of glucokinase, NADPH drain, and feed rate should be varied in order to avoid instability and to find the highest productivity condition. This significantly narrows the focus from 21 variables to just 3.

## Discussion

The results show that EMRA has potential to be a valuable tool for investigating the propensity for stability of complex enzymatic pathways without *a priori* knowledge of specific enzyme parameter values. In three cases presented, (MCC, molecular purge valve, chimeric glycolysis) the experimental investigators were able to heuristically identify productivity issues *ad hoc*, but EMRA is able to unify all these results with a theoretical framework based on instability. Importantly, although some of the phenomena were experimentally determined, it was not necessarily known that instability of the system—causing a step change in the nature of the steady state, rather than a smooth change predictable by sensitivity analysis—could be an underlying reason.

The success of the method with these systems presented here shows that it deserves consideration as a design tool in the invention of new pathways. The method has proven versatile enough to successfully predict features in three different pathways investigated in different laboratories and powerful enough to do so without *a priori* knowledge of specific enzyme parameter values. EMRA simulation of a longer and not-yet characterized pathway demonstrates the range of possibilities for potential applications of this technique. While the characterized pathways were optimized based on intuition, it’s possible that a longer pathway with more enzymes, such as the glucose-to-isoprene pathway, would be much more difficult to optimize without rational balancing methods like those presented here. The reduction of search space from 22 to 3 variables represents an exponentially more approachable experimental path towards productivity, resulting in 27 (3^3^) experiments rather than about 10 million (3^21^) if three different enzyme amounts are tested.

Another insight provided by EMRA and follow-up analysis is the determination of failure modes for the pathways investigated. Using parameter and enzyme amount values in stable and unstable regions of the parameter continuation, time domain integration allows us to determine the failure modes for these pathways upon instability. In the MCC pathway, it is depletion of pathway intermediates—especially X5P, R5P and F6P—which causes productivity decline and eventual stopping. Although time domain simulations weren’t carried out in all systems, the demonstration of failure mechanism in the MCC system may lend credence to the other EMRA examples. In the chimeric glycolysis pathway, depletion of ATP eventually caused that pathway to stop when glucose feed rate was too high. Identifying these failure modes with EMRA is another potentially fruitful area of discovery.

Glycolysis is a fundamental pathway of life and functions successfully in many organisms. However, our simulations and previous experiments (Fig 6, [17]) have shown it can be unstable under high glucose feed conditions which apparently deplete ATP and accumulate hexose-monophosphates (Fig 6D and 6E). Some hexokinase enzymes are product-inhibited by G6P, [22] however, the particular enzyme used in the experimental investigation (from *Thermus thermophilus*) was investigated and no G6P-inhibition was reported.[23] Interestingly, glycolysis has also been shown to be unstable to low levels of inorganic phosphate in yeast, a condition which prevents GAPDH from proceeding [24]. Glycolysis is a nearly universal pathway, but this evidence shows it to be unstable in some cases. This helps to explain the presence of elaborate regulations such as insulin and glucagon [25,26] in animals and the massively sophisticated regulation of phosphofructokinase [27,28] in many organisms. Rather than stability, alternate explanations such as chemical necessity [29] and thermodynamic efficiency [30,31] are more likely reasons for the universality of glycolysis.

In these analyses, EMRA was used to successfully evaluate the stability of complex *cell-free* pathway assays. *In vitro* biocatalysis systems are a powerful alternative and complement to *in vivo* systems [32]. Importantly, however, this does not exclude the possibility of success with simulation of *in vivo* systems. Depending on growth mode (exponential growth, stationary phase, fermentation etc.) *in vivo* systems may have different reference fluxes, so more exploration is required to identify different possibilities.

It is unsurprising that lower amounts of pathway enzymes or feed rate would hinder productivity. The powerful insight provided by these results is that for the pathways identified, increasing levels of certain enzymes or feed rates were shown to cause instability and consequently reduce production. A typical metabolic engineering approach may be to simply maximize the reaction rate of all pathway enzymes. However, we show here that for many enzymes, this will not always result in an optimal outcome.

Additionally, we have shown that the intrinsic stability of pathways varies significantly depending on structure and kinetic forms. This highlights the importance of stability analysis in understanding metabolic systems. Additionally, it shows that many metabolic systems may be very difficult to balance without sufficient rational methods for analyzing which enzymes are most likely to contribute to pathway instability, and in which amounts. This shows the importance of EMRA and stability analysis in general in understanding pathways theoretically and exploiting them practically.

The lack of requirement for *a priori* knowledge of specific enzyme parameter values could make EMRA particularly approachable for experimental researchers working with new pathways or unknown enzymes. This may be hampered somewhat by the need for sophisticated mathematical operations, though this obstacle could be overcome if an appropriate software suite is made available. We believe EMRA can significantly contribute to pathway development efforts and is an important contribution to the toolbox of metabolic engineering.

## Methods

Production systems were described in Systems Description. Stoichiometric matrices for the pathways were constructed based on catalogued and reported reactions. Reversibilities of each enzyme were assigned based on if an enzyme i) is an ATP-dependent kinase ii) catalyzes a reaction with a highly negative ΔG^o^ (less than -20kJ), [33] or iii) catalyzes a decarboxylation. The functional form used for each reaction used canonical Michaelis-Menten saturation kinetics. Affinity parameters (K_m_) are normalized to substrate concentration to reduce the number of parameters sampled for, and metabolite concentrations are normalized to unity. The normalized forms of the equations are as previously shown [7].

For the pathways chosen, exchange reactions were defined for inputs and outputs. Stoichiometric matrices, enzyme reversibilities and reference steady states for all simulations can be found in SI (S1–S7 Tables).

Ensemble modeling robustness analysis (EMRA) was performed on several enzymatic pathways that have been performed experimentally in the literature. EMRA is a technique which uses knowledge of an enzymatic pathway’s network structure to determine if its steady state is stable to changes in enzyme level. Michaelis-Menten style saturation rate equations are determined for each reaction based on stoichiometry and reversibility. Enzyme parameters are picked randomly to satisfy the reference steady state which is provided. For each enzyme, random values in a 100-fold range with a uniform distribution (from 0.1 to 10 fold of the normalized metabolite concentration) were selected for the scaled affinity parameters (K_m_/[X]_ss,r_) [7]. V_max_ is solved for using the reference steady state flux value. To confirm the presence of a stable steady state, the system’s Jacobian is confirmed to have eigenvalues with only negative real parts [34]. Otherwise, the model is discarded. 1,000 parameter sets were generated this way for each system. For the completely random parameter values, a log-uniform 100-fold range of V_max_ parameter was used. For the MCC (Fpk/Xpk 1:3) system, the Fpk and Xpk Vmax were assigned ¼ and ¾, respectively, of the value of a random number generated, to ensure that the overall phosphoketolase amount was controlled. Steady state determination was validated by decreasing integration time by 10-fold, and ensuring that the fraction of 1000 parameter sets which reach steady state was within the SD of the original condition (n = 1000 x 3). V_max_ values were generated on a log-uniform scale, while K_m_ was generated on a uniform scale. To test if method of generating random values was a significant factor, different methods were tested (all values from a uniform distribution or log-uniform distribution) but no major differences were seen (S8 Table).

To simulate batch systems from an analogous continuous system (as in the simulation of the MCC system shown in Fig 4), a minimum of changes were made. Functional forms for reaction flux equations were maintained. For the simulations in Fig 4, an arbitrary parameter set was selected from those generated by ensemble modeling. For all rate law reactions used, reaction rate (V_i_) was directly proportional to V_max_. V_max_ is multiplied by a function of other parameters, ($\bar{\lambda }$ representing K_m,_ K_eq_, etc.) and normalized metabolite concentrations ($\bar{\text{x}}$).

$$V_{i}=V_{max}*f\left(\bar{\lambda },\bar{x}\right)$$

To simulate a batch system, exchange reactions are removed by setting their V_max_ to 0.

Normalized metabolite concentration values were used for both simulation conditions. To provide a supply of starting substrate, the initial amount of formaldehyde was multiplied by 200. Normalized steady state (continuous) or initial (batch) metabolite concentration (${\bar{\text{X}}}_{\text{i}}$) is shown in this table for continuous and batch simulations. Normalized metabolite amount, ${\bar{\text{X}}}_{\text{i}}$, is equal to the actual metabolite concentration, X_i_, divided by the reference steady state concentration, X_i,SS_.

$$\left[{\bar{X}}_{i}\right]=\frac{X_{i}}{X_{i,SS}}$$

In combination, setting input and output V_max_ to 0 and providing starting substrate formaldehyde allows the simulation to run as a batch system accumulating acetate while keeping the other rate equations and parameter values unchanged.

For each parameter set in an ensemble, the steady state is perturbed using parameter-domain integration. This consists of numerically integrating using the continuation method. The mathematical justification for this method has been presented previously [7]. At each step in this integration, as an enzyme level is perturbed, the normalized metabolite concentration at the new steady state may be different, so it is necessary to re-compute the numerical values of the Jacobian matrix. If the new Jacobian has an eigenvalue with a positive real part, this indicates system instability [34] (Fig 1) and the integration is halted. These computations allow for analysis at every level of enzyme perturbation. For each parameter set in an ensemble, the fraction which were stable (i.e. all Jacobian matrix eigenvalues remain negative) can be plotted versus the level of perturbation for each enzyme.

Simulations were performed in MATLAB, and the code required to reproduce all figures is available at: https://github.com/theis188/theisen-plos-comp-bio.

## Supporting Information

S1 Fig EMRA stability profiles for canonical EMP glycolysis converting glucose to lactate with an ATP drain (ATPD). (TIF)

S1 Table S, Vref and reversibilities of enzymes for Fpk version of MCC. (DOCX)

S2 Table S, Vref and reversibilities of enzymes for Xpk version of MCC. (DOCX)

S3 Table S, Vref and reversibilities of enzymes for X/Fpk version of MCC. (DOCX)

S4 Table S, Vref and reversibilities of enzymes for chimeric glycolysis pathway. (DOCX)

S5 Table S, Vref and reversibilities of enzymes for EMP glycolysis pathway with ATP drain. (DOCX)

S6 Table S, Vref and reversibilities of enzymes for molecular purge pathway. (DOCX)

S7 Table S, Vref and reversibilities of enzymes for glucose-to-isoprene pathway. (DOCX)

S8 Table Comparison of different methods for determining random values from Fig 3A. (DOCX)
