# Supplementary material for: Stability of Ensemble Models Predicts Productivity of Enzymatic Systems
Source: PLoS Comput Biol. 2016 Mar 10;12(3):e1004800. doi: 10.1371/journal.pcbi.1004800 (PMC4786283; doi:10.1371/journal.pcbi.1004800)
Supplement: S3 Table — (DOCX) [file pcbi.1004800.s004.docx]

|  | In | Hps | Phi | Tkt | Ttk | Tal | Fpk | Xpk | Rpe | Rpi | Out |
| --- | --- | --- | --- | --- | --- | --- | --- | --- | --- | --- | --- |
| Reversibilities | 1 | 1 | 1 | 1 | 1 | 1 | 0 | 0 | 1 | 1 | 0 |
| Vref | 4 | 4 | 4 | 2 | 1.5 | 2 | 0.5 | 1.5 | 2 | 2 | 2 |
| MeO | 1 | -1 | 0 | 0 | 0 | 0 | 0 | 0 | 0 | 0 | 0 |
| Ru5P | 0 | -1 | 0 | 0 | 0 | 0 | 0 | 0 | 1 | 1 | 0 |
| H6P | 0 | 1 | -1 | 0 | 0 | 0 | 0 | 0 | 0 | 0 | 0 |
| F6P | 0 | 0 | 1 | 0 | -1 | -1 | -1 | 0 | 0 | 0 | 0 |
| E4P | 0 | 0 | 0 | 0 | 1 | -1 | 1 | 0 | 0 | 0 | 0 |
| G3P | 0 | 0 | 0 | -1 | -1 | 1 | 0 | 1 | 0 | 0 | 0 |
| S7P | 0 | 0 | 0 | -1 | 0 | 1 | 0 | 0 | 0 | 0 | 0 |
| X5P | 0 | 0 | 0 | 1 | 1 | 0 | 0 | -1 | -1 | 0 | 0 |
| R5P | 0 | 0 | 0 | 1 | 0 | 0 | 0 | 0 | 0 | -1 | 0 |
| AcP | 0 | 0 | 0 | 0 | 0 | 0 | 1 | 1 | 0 | 0 | -1 |

Table S3. **S, Vref** and reversibilities of enzymes for X/Fpk version of MCC.
