# Supplementary material for: Stability of Ensemble Models Predicts Productivity of Enzymatic Systems
Source: PLoS Comput Biol. 2016 Mar 10;12(3):e1004800. doi: 10.1371/journal.pcbi.1004800 (PMC4786283; doi:10.1371/journal.pcbi.1004800)
Supplement: S4 Table — (DOCX) [file pcbi.1004800.s005.docx]

|  | GK | PGI | PFK | ALD | TPI | GAPN | PGM | ENL | PYK | LDH | IN | OUT |
| --- | --- | --- | --- | --- | --- | --- | --- | --- | --- | --- | --- | --- |
| Reversibilities | 0 | 1 | 0 | 1 | 1 | 1 | 1 | 1 | 0 | 0 | 1 | 0 |
| Vref | 1 | 1 | 1 | 1 | 1 | 2 | 2 | 2 | 2 | 2 | 1 | 2 |
| Glc | -1 | 0 | 0 | 0 | 0 | 0 | 0 | 0 | 0 | 0 | 1 | 0 |
| G6P | 1 | -1 | 0 | 0 | 0 | 0 | 0 | 0 | 0 | 0 | 0 | 0 |
| F6P | 0 | 1 | -1 | 0 | 0 | 0 | 0 | 0 | 0 | 0 | 0 | 0 |
| FBP | 0 | 0 | 1 | -1 | 0 | 0 | 0 | 0 | 0 | 0 | 0 | 0 |
| G3P | 0 | 0 | 0 | 1 | -1 | 0 | 0 | 0 | 0 | 0 | 0 | 0 |
| DHAP | 0 | 0 | 0 | 1 | 1 | -1 | 0 | 0 | 0 | 0 | 0 | 0 |
| 3PG | 0 | 0 | 0 | 0 | 0 | 1 | -1 | 0 | 0 | 0 | 0 | 0 |
| 2PG | 0 | 0 | 0 | 0 | 0 | 0 | 1 | -1 | 0 | 0 | 0 | 0 |
| PEP | 0 | 0 | 0 | 0 | 0 | 0 | 0 | 1 | -1 | 0 | 0 | 0 |
| Pyr | 0 | 0 | 0 | 0 | 0 | 0 | 0 | 0 | 1 | -1 | 0 | 0 |
| ATP | -1 | 0 | -1 | 0 | 0 | 0 | 0 | 0 | 1 | 0 | 0 | 0 |
| ADP | 1 | 0 | 1 | 0 | 0 | 0 | 0 | 0 | -1 | 0 | 0 | 0 |
| NAD | 0 | 0 | 0 | 0 | 0 | -1 | 0 | 0 | 0 | 1 | 0 | 0 |
| NADH | 0 | 0 | 0 | 0 | 0 | 1 | 0 | 0 | 0 | -1 | 0 | 0 |
| Lac | 0 | 0 | 0 | 0 | 0 | 0 | 0 | 0 | 0 | 1 | 0 | -1 |

Table S4. **S, Vref** and reversibilities of enzymes for chimeric glycolysis pathway.
