# Supplementary material for: Stability of Ensemble Models Predicts Productivity of Enzymatic Systems
Source: PLoS Comput Biol. 2016 Mar 10;12(3):e1004800. doi: 10.1371/journal.pcbi.1004800 (PMC4786283; doi:10.1371/journal.pcbi.1004800)
Supplement: S6 Table — (DOCX) [file pcbi.1004800.s007.docx]

|  | 'PDHP' | 'PDHN' | 'NoxE' | 'AtoB' | 'Hbd' | 'IN' | 'OUT' |
| --- | --- | --- | --- | --- | --- | --- | --- |
| Reversibilities | 0 | 0 | 0 | 1 | 1 | 1 | 0 |
| Vref | 0.5 | 0.5 | 0.5 | 0.5 | 0.5 | 1 | 0.5 |
| 'Pyr' | -1 | -1 | 0 | 0 | 0 | 1 | 0 |
| 'AcCoA' | 1 | 1 | 0 | -2 | 0 | 0 | 0 |
| 'AcAcCoA' | 0 | 0 | 0 | 1 | -1 | 0 | 0 |
| 'BuCoA' | 0 | 0 | 0 | 0 | 1 | 0 | -1 |
| 'NADH' | 0 | 1 | -1 | 0 | 0 | 0 | 0 |
| 'NAD' | 0 | -1 | 1 | 0 | 0 | 0 | 0 |
| 'NADPH' | 1 | 0 | 0 | 0 | -1 | 0 | 0 |
| 'NADP' | -1 | 0 | 0 | 0 | 1 | 0 | 0 |

Table S6. **S, Vref** and reversibilities of enzymes for molecular purge pathway.
