# Supplementary material for: Stability of Ensemble Models Predicts Productivity of Enzymatic Systems
Source: PLoS Comput Biol. 2016 Mar 10;12(3):e1004800. doi: 10.1371/journal.pcbi.1004800 (PMC4786283; doi:10.1371/journal.pcbi.1004800)
Supplement: S8 Table — (DOCX) [file pcbi.1004800.s009.docx]

**Table S8.** Comparison of different methods for determining random values from Fig. 3a.

|  | MCC  (Fpk only) | MCC  (Xpk only) | MCC (F/Xpk 1:3) | Molecular Purge Valve | Chimeric Glycolysis | Gluc to Isoprene (With Regulation) | Gluc to Isoprene |
| --- | --- | --- | --- | --- | --- | --- | --- |
| Vmax Log,  Km Uniform | 43% | 27% | 19% | 49% | 58% | 36% | 21% |
| All Linear | 40% | 23% | 14% | 49% | 59% | 43% | 27% |
| All Log | 36% | 26% | 18% | 49% | 57% | 37% | 21% |
